# Comparative embryology of *Delphinapterus leucas* (beluga whale), *Balaena mysticetus* (bowhead whale), and *Stenella attenuata* (pan‐tropical spotted dolphin) (Cetacea: Mammalia)

**DOI:** 10.1002/jmor.21543

**Published:** 2023-01-16

**Authors:** Lia Gavazzi, Lisa N. Cooper, Sharon Usip, Robert Suydam, Raphaela Stimmelmayr, John C. George, Greg O'Corry‐Crowe, Chih‐Wei Hsu, Johannes Thewissen

**Affiliations:** ^1^ School of Biomedical Sciences Kent State University Kent Ohio USA; ^2^ Musculoskeletal Research Focus Area, Department of Anatomy and Neurobiology Northeast Ohio Medical University Rootstown Ohio USA; ^3^ Department of Wildlife Management North Slope Borough Barrow Alaska USA; ^4^ Institute of Arctic Biology University of Alaska Fairbanks Fairbanks Alaska USA; ^5^ Harbor Branch Oceanographic Institute Florida Atlantic University Fort Pierce Florida USA; ^6^ Department of Molecular Physiology and Biophysics Baylor College of Medicine Houston Texas USA

**Keywords:** development, evolution, heterochrony, land‐to‐sea transition

## Abstract

Embryogenesis of cetaceans (whales, dolphins, porpoises) is best known in *Stenella attenuata*, the pan‐tropical spotted dolphin, based on a remarkably complete and well‐studied prenatal ontogenetic series. Our study expands understanding of cetacean embryology by adding two additional cetacean taxa: the beluga whale (*Delphinapterus leucas*, Odontoceti), and the bowhead whale (*Balaena mysticetus*, Mysticeti). We identify key features that characterize these taxa at specific stages and highlight heterochrony between the odontocetes and mysticetes. The toothed whales are more similar in developmental timing to each other than either is to *Balaena*. The two odontocete taxa, *Stenella* and *Delphinapterus*, share similar developmental trajectories while early *Balaena* specimens differ from the odontocetes. This developmental variation proves challenging to ascribe to the existing Carnegie staging system. Most notably, flippers, hindlimbs, and flukes all provide morphological traits for characterization within the Carnegie staging system. A presomitic *Delphinapterus* embryo is also described. This study applies the Carnegie staging system to two more cetacean taxa and forms a framework for future research on cetacean developmental genetics and the modeling of fetal growth.

## Graphical abstract

Cetaceans both depart from and are defined by their mammalian biology. This paradox is best exemplified during embryonic development. Here, we discuss stages of prenatal development in the beluga whale (*Delphinapterus leucas*), the bowhead whale (*Balaena mysticetus*), and the pan‐tropical spotted dolphin (*Stenella attenuata*), calling attention to heterochronic variation found between all three taxa.

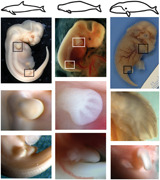

## 1 INTRODUCTION

Nearly every aspect of cetacean biology is modified to accommodate their exclusively aquatic lifestyles. This transition from land to water is a remarkable feat, considering that these taxa are ultimately constrained by the mammalian bauplan and still demonstrate classic mammalian features, such as live birth, nursing, homeothermy, and oxygen intake via nares to lungs. Early during the embryonic period, cetaceans appear similar to other mammals with processes like somite formation, forelimb bud development, and patterning of organ primordia (Štĕrba et al., 2000; Thewissen & Heyning, 2007). As development progresses, these embryos begin to diverge from this mammotypical plan and take on cetacean‐specific traits including loss of external hindlimbs (Guldberg & Nansen, 1894; Ogawa, 1953; Sedmera et al., 1997a; Thewissen et al., 2006), the formation of a soft‐tissue fluke for tail‐based propulsion (Buchholtz, 2007; Fish, 1998; Ryder, 1885; Thewissen, 2018), blowhole formation on the dorsum of the skull (Farnkopf et al., 2021; Haddad et al., 2012; H. H. A. Oelschläger, 2000; Roston & Roth, 2019, 2021), and hyperphalangy (Cooper et al., 2007, 2011, 2017; Richardson & Chipman, 2003; Richardson et al., 2009; Richardson & Oelschlager, 2002; Sedmera et al., 1997b). Although other aquatic mammals such as Sirenia (manatees and dugongs) also lack external hindlimbs and display adaptations for tail‐powered swimming (Buchholtz et al., 2007; Domning, 2018), only cetaceans evolved a blowhole on the top of their heads and hyperphalangy. Hyperphalangy is defined as an increase in the number of phalanges from the standard mammalian phalangeal formula, from anterior to posterior, of 2/3/3/3/3 (Cooper et al., 2007; Fedak & Hall, 2004; Richardson & Chipman, 2003; Richardson et al., 2009; Richardson & Oelschlager, 2002). Variation in phalangeal count in cetacean taxa is often both intra‐ and interspecific (Cooper et al., 2007; Sedmera et al., 1997b).

Furthermore, most odontocetes (dolphins and porpoises) have more teeth than is typical for mammals and have developed homodonty (Armfield et al., 2013), whereas beaked whales have fewer, specialized teeth, and baleen whales (mysticetes) have replaced their dentition with keratin‐based baleen plates, a novel adaptation for filter feeding (Lanzetti et al., 2020; Marx et al., 2017; Pivorunas, 1979; Thewissen et al., 2017).

The cetacean taxon for which ontogeny is best described is *Stenella attenuata*, the pan‐tropical spotted dolphin. A collection of prenatal specimens curated and housed at the Natural History Museums of Los Angeles County illuminates almost the entirety of organogenesis and has been utilized in numerous studies of cetacean embryonic morphology (Armfield et al., 2011; Moran et al., 2011; H. H. A. Oelschläger, 2000; Richardson & Oelschlager, 2002; Roston et al., 2013; Roston & Roth, 2021; Sedmera et al., 1997a, 1997b, 2003; Štĕrba et al., 2000; Thewissen et al., 2006; Thewissen & Heyning, 2007). Furthermore, protein signaling studies have been conducted using this *Stenella* collection (Armfield et al., 2013; Cooper et al., 2017; Thewissen et al., 2006, 2017). The nearly complete range of developmental ages makes this collection unique, and thus forms the basis for our understanding of cetacean embryology and evolutionary developmental biology.

Other knowledge about prenatal cetaceans is based on material from commercial whaling operations (Guldberg & Nansen, 1894; Kükenthal, 1889; Ogawa, 1953; Roston et al., 2013; Roston & Roth, 2021; Ryder, 1885; Stump et al., 1960), collaboration with indigenous communities that harvest cetaceans for subsistence (Armfield et al., 2011; Farnkopf et al., 2021; Heide‐Jørgensen & Garde, 2011; Thewissen et al., 2017), strandings (Berta et al., 2015; Roston & Roth, 2021), and from cetacean bycatch during commercial fishing (Armfield et al., 2011; Cooper et al., 2017; Moran et al., 2011; Roston & Roth, 2021; Sedmera et al., 1997a, 2003; Štĕrba et al., 2000; Thewissen et al., 2006; Thewissen & Heyning, 2007). Many of these reports focus on morphological descriptions of fetal specimens. These fetal specimens answer important questions of allometry and scaling during gestation, particularly as there is access to multiple specimens of the same taxon that span an ontogenetic range (Haddad et al., 2012; Hampe et al., 2015; Heide‐Jørgensen & Garde, 2011; Lanzetti et al., 2020; Roston et al., 2013; Roston & Roth, 2021). However, most features that define Cetacea, such as loss of external hindlimbs, fluke outgrowth, and hyperphalangy, are established earlier during the embryonic period, where samples or detailed studies are limited. Additionally, prenatal specimens from stranded pregnant females are usually too poorly preserved for use in developmental research as the RNA and proteins have degraded. Given the dramatic morphologies found in cetaceans, there is a need for a better understanding of the molecular mechanisms that underpin these traits.

This study describes major features of critical stages of embryonic development in the beluga whale (*Delphinapterus leucas*) and the bowhead whale (*Balaena mysticetus*) taken during legal subsistence harvests in Alaska. Additionally, we describe a presomitic *Delphinapterus* specimen and compare our findings to other reports on early‐stage cetacean embryos (Asada et al., 2001; Stump et al., 1960). We place these two taxa within the pre‐existing cetacean Carnegie staging system developed by Thewissen and Heyning (2007). We highlight specific ontogenetic comparisons between multiple taxa during the embryonic period, notably phalangeal count, the timing of hindlimb loss, and fluke outgrowth from the tail. We discuss major transitions during embryonic development, divergences between mysticetes and odontocetes, and consider heterochronic shifts between the three taxa. We hypothesize a high level of similarity in the relative developmental timing of the characters described here between the odontocetes. Thus, we anticipate that *Balaena* will show the greatest degree of heterochronic variation when compared to *Stenella*. Our data demonstrate the importance of utilizing externally visible characters, not just total length or weight, as metrics of developmental staging.

## 2 MATERIALS AND METHODS

We examined the external morphology of embryonic and fetal specimens of three cetaceans: *D. leucas*, *B. mysticetus*, and *S. attenuata*. Morphological features used here to define different Carnegie stages (CSs) have been previously identified and categorized for *S. attenuata* (Štĕrba et al., 2000; Thewissen & Heyning, 2007). Štĕrba et al. (2000) utilized a few embryonic specimens of *Delphinus delphis*, the common dolphin, and *Phocoena phocoena*, the harbor porpoise, in their staging system as well. All embryos and fetuses referenced herein are currently housed at the Northeast Ohio Medical University in Rootstown, OH, USA.

The referenced collection of *Stenella* embryos was drawn from the Natural History Museums of Los Angeles County (LACM), and, before that, was housed at the National Oceanic and Atmospheric Administration in La Jolla, CA. These *Stenella* embryos were collected as incidental bycatch of the tuna fishing industry in the north Pacific in the 1970s. Both Štĕrba et al. (2000) and Thewissen and Heyning (2007) used a portion of this collection to generate their respective staging systems. This collection provides the most complete ontogenetic series of the three taxa referenced here, covering early somitogenesis to fetal development.

In northern Alaska, Iñupiat and Siberian Yupik communities from 11 whaling communities legally harvest *Balaena* on their seasonal migration between the Bering and Beaufort sea during the spring and fall (Suydam & George, 2021). Pregnant females are occasionally harvested unintentionally, carrying an embryo or fetus. Given that mating appears to be restricted to a short period in March or early April and the gestation of *Balaena* is 13–14 months (Reese et al., 2001; Tarpley et al., 2021), prenatal *Balaena* specimens obtained during these two seasonal harvesting periods include the embryo‐fetus transition, mid‐gestation, and perinatal fetuses. Collection of samples was led by the North Slope Borough Department of Wildlife Management (NSB‐DWM) in collaboration with the Alaska Eskimo Whaling Commission. All specimens were collected under NOAA‐NMFS permit 17350.

Iñupiat residents from Point Lay, Alaska also legally harvest *Delphinapterus* for subsistence purposes. The subsistence harvest typically occurs between late‐June and mid‐July with the migration of *Delphinapterus* within the Kasegaluk Lagoon. Similar to *Balaena*, only specific age ranges of embryos and fetuses can be collected due to the timing of mating and harvest: around the embryo‐fetal transition and perinatal, given that *Delphinapterus* gestation lasts approximately 13 months (Suydam, 2009). The *Delphinapterus* embryos and fetuses described were collected under NOAA NMFS permit 17350 under the lead of the NSB‐DWM in collaboration with Point Lay. The collection of embryonic material for both taxa is made possible through the continued support, generosity, and hospitality of the communities of Point Lay and Utqiaġvik, Alaska during annual harvests. This work would not be possible without the cooperation of the subsistence hunters.

The NSB‐DWM ID system uses abbreviations to refer to the village in which the embryo was recovered. Within *D. leucas*, the addition of DL indicates the taxon to distinguish from *Balaena*. The addition of an F at the end differentiates between the mother and the fetus (F).

All *Stenella* specimens references herein were preserved in 70% ethanol at ambient temperature over several decades before being transferred to NEOMED. These specimens are currently stored in fresh 70% ethanol at 4°C. The Alaskan cetacean specimens, *Delphinapterus* and *Balaena*, were initially fixed in fresh‐made 4% PFA mixed with seawater before being transferred to 70% ethanol for long‐term storage at 4°C.

To document and describe a presomitic *Delphinapterus* specimen, (NSB‐DWM 2017LDL21F), we used a modified dice‐CT protocol specific for embryonic tissue (Hsu et al., 2016, 2019). The specimen was immersed in iodine overnight before being mounted and embedded in low melt agarose. This agarose block was scanned on a Bruker SkyScan 1272 micro‐CT Scanner at 2 μ at the Baylor College of Medicine Optical Imaging and Vital Microscopy Core in Houston, TX. Iodine staining before micro‐CT scanning allows for visible contrast and differentiation between soft tissue structures. Additionally, iodine is removable from the tissue without causing permanent damage, allowing for additional analysis via other methods. Using this micro‐CT data, NSB‐DWM 2017LDL21F was virtually segmented for analysis of the internal structure using Avizo 2019.4 (ThermoFisher). After scans were completed, the specimen was extracted from the agarose gel and immersed in sodium thiosulfate to remove iodine. The specimen was then further prepared for paraffin‐sectioned histology and cut in 6‐μ thick sections, mounted on glass slides, and stained using hematoxylin and eosin.

All specimens described herein were referred to CSs based on the criteria of the *Stenella* staging system (Thewissen & Heyning, 2007). With one exception (the presomitic *Delphinapterus* embryo), the specimens pertain to CS 16–21. All specimen numbers, lengths, and weights are recorded in Table 1.

**Table 1:** Summary of the *Delphinapterus*, *Balaena*, and *Stenella* embryo collection

	Stage	CRL/TL (mm)	Weight (g)
*Delphinapterus* ID (NSB‐DWM)			
2017LDL21F	PS	2	U
2019LDL15F	16	33*	1.2
2013LDL6F	17	67*	2.1
2009LDL17F	19	90*	4.1
2011LDL11F	19	93	6.1
2009LDL9F	19	93	U
2019LDL25F	20	134	12.3
2013LDL4F	20	136	19.5
2013LDL21F	20	139	20.1
2009LDL12F	20	139	19.3
2017LDL3F	20	153	U
2014LDL4F	20	156	39.7
2014LDL5F	20	180	46
2012LDL3F	F	184	64.3
2014LDL7F	F	187	70.1
2012LDL10F	F	197	U
2019LDL11F	F	198	60.5
2012LDL9F	F	200	61.4
2016BDL3F	F	231	131.2
2012BDL1F	F	255	157.6
*Balaena* ID (NSB‐DWM)			
1999B7F	17	87	U
2018G3F	19	90	4.6
2016B9F	20	128	25
1999B6F	20	166	U
2013B1F	21	274	U
2000B3F	21	403	U
2007B16F	23	1590	U
2009KK1F	23	1630	U
2015B9F	23	4220	U
*Stenella* ID (LACM)			
94756	16	11*	
94601	16	15*	
94745	17	17*	
94820	17	19*	
94673	17	20*	
94717	18	21*	
94696	19	30*	
94743	19	34*	
95051	19	30*	
90310	20	U	
94646	20	103	
95014	20	116	
94676	20	117	
94419	21/22	160	
94413	21/22	151	
94421	23	252	

*Note*: Samples are either on loan from the North Slope Borough Department of Wildlife Management (NSB‐DWM) or from the Natural History Museums of Los Angeles County (LACM). Within *Delphinapterus leucas*, the addition of DL indicates the taxon to distinguish from *Balaena*. All measurements rounded to nearest millimeter or nearest tenth of a gram. Measurement of crown‐rump length (CRL) indicated with an asterisk (*), total length (TL) indicated otherwise. Weight is wet weight after fixation. Unknown data points indicated with U.

Abbreviations: B, Barrow (Utqiaġvik); F, fetus; G, Gambell; KK, Kaktovik, Alaska; L, Point Lay.

## 3 RESULTS

### 3.1 NSB‐DWM 2017LDL21F, a presomitic specimen of *D. leucas*

*Delphinapterus* specimen NSB‐DWM 2017LDL21F was collected from a sexually mature female. One ovary had a corpus luteum, indicating pregnancy. The embryo has an oval shape, approximately 2 mm in length and 1 mm in width, with a short curved process and extensive fetal membranes. These membranes were not embedded within the endometrium of the mother's uterus.

#### 3.1.1 Fetal membranes

**Figure 1:**
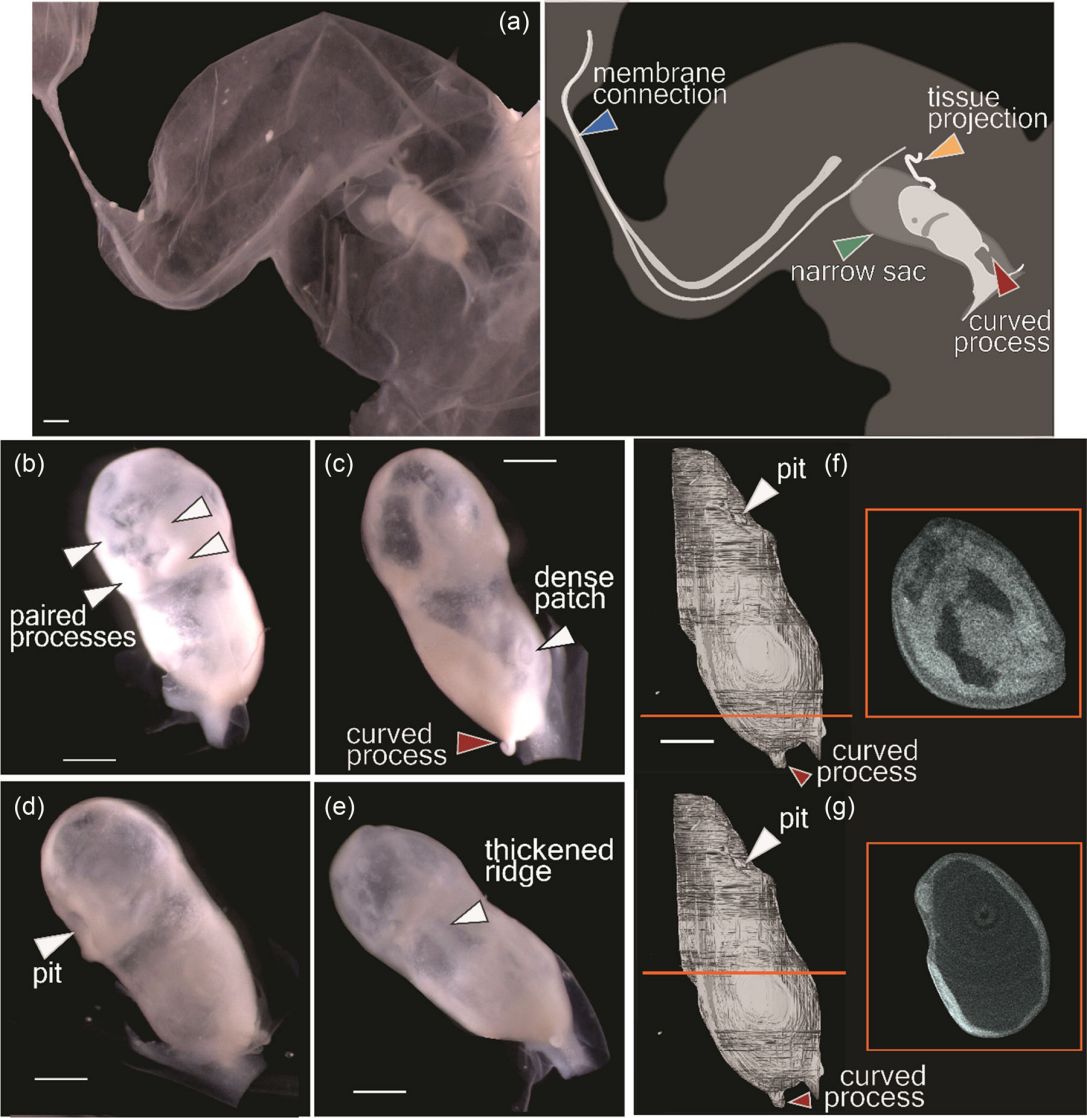
Photographs (a–e) and micro‐CT images (f and g) of the presomitic *Delphinapterus* embryo (NSB‐DWM 2017LDL21F). (a) Specimen with supporting membranes and explanatory diagram. (b–e) Photographs of embryo with membranes removed. (f and g) Micro‐CT reconstructions with cross‐sectional morphology. Orange line indicates plane of section. Scale bar equals 1 mm.

The membranes surrounding NSB‐DWM 2017LDL21F (Figure 1a) are attached to one of the narrow ends of the specimen, at the same end as the curved process (red arrow). A narrow sac surrounds the embryo (Figure 1a, green arrow), and extraembryonic membranes surround both structures. A long, thin curled strip of tissue projects from this smaller sac (Figure 1a, orange arrow). Attached to the large extraembryonic membranes are two smaller pouches. Their attachment is by a narrow strip of opaque tissue (Figure 1a, blue arrow). All of the external supporting membranes are similar in consistency.

#### 3.1.2 External morphology

The most prominent feature found on this specimen is the small, curved process (Figure 1c, red arrow) on one end of the embryo. In addition, there are two paired processes (Figure 1b) on the flat surface opposite of the curve process. These encircle a small pit (Figure 1d). Near these processes are two dense patches, one of which is visible in Figure 1c (arrow). On the surface opposite the paired projections, a thickened ridge of tissue occurs that may represent the presumptive midline of the specimen (Figure 1e, arrow).

#### 3.1.3 Internal morphology

Virtual segmentation of the micro‐CT data reveals that the specimen has an internal cavity that is divided into regions. Toward the end of the specimen with the curled projection, two smaller chambers are separated by a thin septum. These chambers are a bilaterally symmetrical space with a small tissue projection into the center (Figure 1f). These chambers are not connected to a second space that constitutes the majority of the internal structure of the specimen (Figure 1g).

#### 3.1.4 Histology

We describe four representative slides from this *Delphinapterus* specimen.

**Figure 2:**
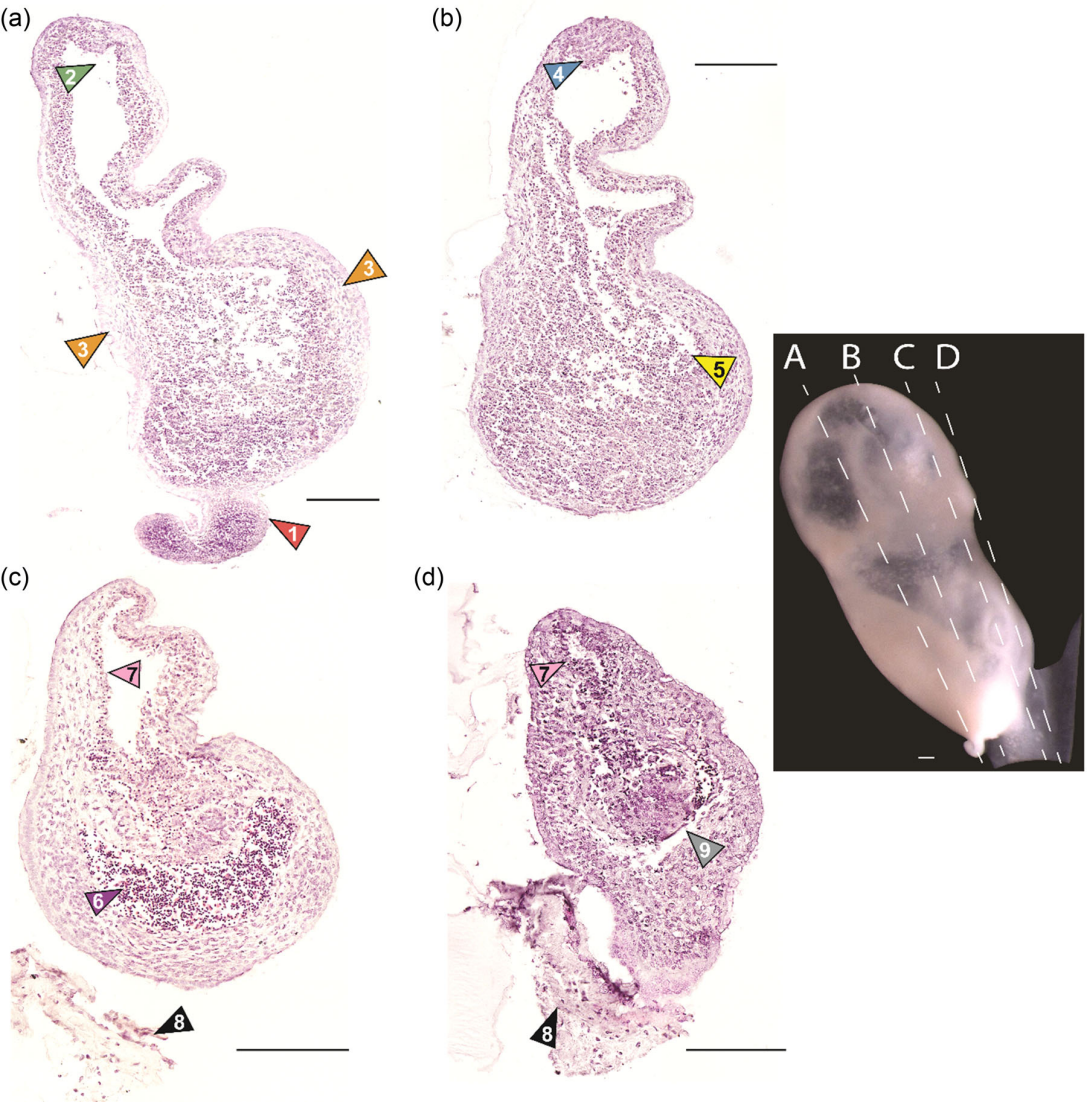
Histological sections for presomitic *Delphinapterus* embryo (NSB‐DWM 2017LDL21F). Sections 6‐μ thick and stained with hematoxylin and eosin. Scale bars equal 200 μm. (a) 1, red—curved projection; 2, green—lumen; 3, orange—mesenchymal tissue. (b) 4, blue—tissue projecting into lumen; 5, yellow—erythrocytes. (c) 6, purple—erythrocyte region; 7, pink—connective tissue lining lumen; 8, black—extraembryonic membranes. (d) 9, gray arrow—glandular cells. Right: A photograph of the embryo with dashed lines indicating the approximate plane of section for each histological image.

Figure 2a—This section shows the curved process attached to the narrow end of the embryo (1, red arrow). This hypercellular region has a little extracellular matrix. A large lumen is present in the main body of the specimen in this section (2, green arrow). This lumen is lined by a simple epithelium that is cuboidal or columnar in appearance. Nearly all of the cells in this outer layer are vacuolated. A connective tissue surrounds the lumen of the cavity; there is no endothelium. Two patches of circumferentially arranged mesenchymal tissue occur on the sides, with nuclei that are more dispersed compared to surrounding tissue and there are larger swaths of extracellular matrix (3, orange arrows).

Figure 2b—The large cavity described in Figure 2a is also visible in this slide. The section is predominantly comprised of large regions of undifferentiated connective tissue. There is a small region of connective tissue that projects back into the lumen (4, blue arrow). As in Figure 2a, there is no epithelium lining the internal cavity. The internal connective tissue is hypercellular and shows no obvious architecture or differentiation. Patches of erythrocytes are scattered throughout the internal region (5, yellow arrow).

Figure 2c—Unlike previous sections, some anatomical structures are apparent in this section. The most obvious is a large crescent‐shaped region dense with erythrocytes that lacks an endothelial layer (6, purple arrow). This area may represent a cardiogenic region. The undifferentiated, patchy connective tissue in Figure 2a,b is replaced by a connective tissue internal to the blood‐filled region. The connective tissue in this section has a patchy organization compared to the connective tissue seen in previous sections. External to the blood region are several layers of circumferentially arranged mesenchymal cells that appear morphologically similar to the two small lateral patches found in Figure 2b. In the area surrounding the lumen, the morphology of the cells is different from previous sections. The connective tissue surrounding the lumen in Figure 2c is more diffuse, with more extracellular matrix between cells. In Figure 2b, this area was filled with cells that were homogenous and undifferentiated; in this section, there are differences between the mesenchyme and a small, densely cellular connective tissue layer that lines the cavity (7, pink arrow). Neither connective tissue layer has an endothelial layer. The appearance of this densely cellular connective tissue is similar to the hypercellular regions found in Figure 2a,b. In this section, a simple columnar epithelium encircles the different connective tissues, with some areas demonstrating a simple squamous morphology. No basement membrane is apparent between the epithelial and connective tissue layers. Some of the extraembryonic membranes (8, black arrow) are visible, although they do not attach directly to the specimen in this section.

Figure 2d—There is a centrally located patch within this section that is distinct from any other tissue observed thus far and is glandular in appearance (9, gray arrow). In previous sections, there is a large lumen. In this section, this space is reduced in size, and it is surrounded by a hypercellular connective tissue with little extracellular matrix and no corresponding endothelium (7, pink arrow). This region appears to be more densely populated with cells than the corresponding layer in Figure 2c. The epithelium is predominantly squamous in this section, and most of the cells have vacuolated nuclei similar to Figure 2a. Most of the connective tissue is mesenchymal in appearance, although more densely packed than Figure 2c. The large blood‐filled crescent is now reduced to a few small patches surrounding this glandular circle. The connection between the specimen and extraembryonic membranes is visible here (8, black arrow). At this connecting point, nuclei from the specimen appear to be circumferentially oriented around the attachment to the membranes. The membranes themselves are largely comprised of extracellular matrix with a few nuclei that do not appear to be arranged. There is no epithelial tissue in this region.

### 3.2 Comparative embryogenesis of *Delphinapterus*, *Balaena*, and *Stenella*

#### 3.2.1 Stage 16

CS 16 is characterized in *S. attenuata* by the absence of branchial clefts, and presence of a handplate, and eye pigmentation (Thewissen & Heyning, 2007).

##### Delphinapterus

**Figure 3:**
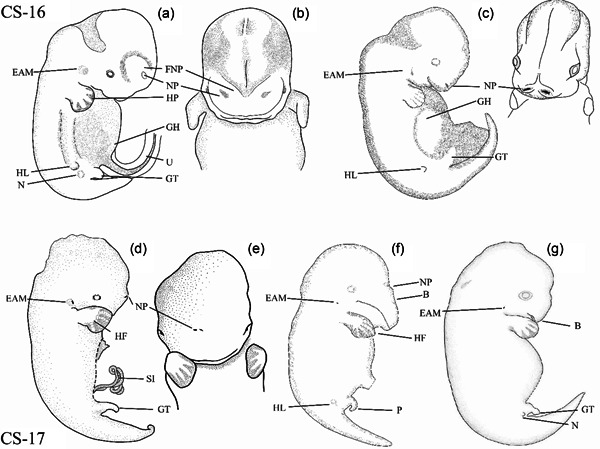
Embryos of *Delphinapterus* (a, b, d, e), *Balaena* (f), and *Stenella* (c, g) for Carnegie stages 16 and 17. All *Delphinapterus* specimens shown in left lateral and frontal view. All *Balaena* and *Stenella* specimens shown in left lateral view. (a and b) NSB‐DWM 2019LDL15F. (c) LACM 94756. (d and e) NSB‐DWM 2013LDL6F. (f) NSB‐DWM 1999B7F, (g) LACM 94673. B, beak; EAM, external acoustic meatus; FNP, frontonasal process; GH, gut herniation, GT, genital tubercle; HF, hyperphalangeous flipper; HL, hindlimb; HP, handplate; NP, nasal pits; SI, small intestine; P, penis; U, umbilicus. Not to scale.

In *Delphinapterus* specimen NSB‐DWM 2019LDL15F (Figure 3a), there is a low eminence located just lateral to the genitals that we hypothesize is the hindlimb bud. This hindlimb bud has a pointed protrusion on the outermost edge of the structure. The gut is herniated into the umbilical cord. Just beneath the hernia, a small genital tubercle is present. The tail is elongated with no lateral outgrowths or indication of fluke formation. There is clear eye pigmentation and no formation of eyelids (Figure 3b). The nasal placodes are relatively large and divided within the fused medial and lateral frontonasal prominences. The left and right nasal prominences are located far from the midline and are located rostrally on the head, which is typical for mammalian development at this stage. The external acoustic meatus is marked by a small posterior prominence.

##### Comparisons with *Stenella*

Like the stage 16 *Stenella* embryo (Figure 3c), the *Delphinapterus* specimen (NSB‐DWM 2019LDL15F) has eye pigmentation, no branchial clefts, and a handplate. The *Delphinapterus* handplate is mediolaterally expanded, dorsoventrally flat with digital rays with no individual phalangeal segments. This morphology is reached at a later stage in *Stenella*, which do not form digital rays until CS 17. The digital rays of *Delphinapterus* are present as anlagen. Both *Delphinapterus* and *Stenella* embryos at CS 16 have hindlimb buds. In *Stenella*, this is the stage where hindlimb buds begin to regress.

#### 3.2.2 Stage 17

CS 17 in *Stenella* is defined by digital ray formation, a digit III that is longer than digit II, and initiation of fluke outgrowth (Thewissen & Heyning, 2007).

##### Delphinapterus

In this *Delphinapterus* specimen (NSB‐DWM 2013LDL6F, Figure 3d), individual digit rays are visible within the flipper and the forelimb shows slight differential outgrowth of digits, with digit III being nearly equal in length to digit II. Hyperphalangy is present in this specimen and readily visible with five individual phalanges visible in digits III and IV. The presence of hindlimb buds cannot be determined due to damage to the abdomen. Loops of small intestine protrude into the umbilical cord, as they do in later embryos. This is the first stage that shows initiation of lateral fluke outgrowths, appearing as a small diamond. The eye is pigmented with no eyelid (Figure 3e). A small hillock is located posterior to the external acoustic meatus. Left and right nasal pits are separated in the midline from each other, though greatly reduced in size in comparison to the CS 16 *Delphinapterus* specimen, NSB‐DWM 2019LDL15F.

##### Balaena

This *Balaena* specimen (NSB‐DWM 1999B7F, Figure 3f) has a pointed flipper with segmentation of the individual phalanges. Digits II and IV have four phalanges while digit III has five. Digit III is also longer than digit II. The intestines are herniated into the umbilical cord. The hindlimb bud is small and round. There are small fluke outgrowths budding off the tail, creating a slight diamond shape (Figure 8). The eye has no visible pigmentation, and the eyelids are fused. There is a large auricular hillock present, with a smaller hillock just superior to it. The external acoustic meatus is located just posterior to these hillocks. Along the curved rostrum, there is a distinct notch for the nasal pits, which are unfused along the midline.

##### Comparisons with *Stenella*

The flipper in this *Delphinapterus* specimen (Figure 3a) is more developed than the *Stenella* flipper (Figure 3g). The *Delphinapterus* digits are segmented, and digits II and IV are hyperphalangeous, while the *Stenella* digital rays have not yet segmented. All other features mentioned for *Delphinapterus* are similar to *Stenella* embryos of this stage, including most notably fluke outgrowth (Figure 7).

Like the *Delphinapterus* embryo, the *Balaena* flipper has a more developed morphology, with hyperphalangy already clearly present. Hyperphalangy is present in digits II, III, and IV in *Balaena*. At this stage, digits II and IV have equal numbers of phalanges in *Delphinapterus*. Digit III of *Balaena* has the greatest number of phalanges for that taxon at five while the other hyperphalangeous digits, digits II and IV, have four phalanges. While both the *Balaena* and *Stenella* have a visible beak at this stage, the *Balaena* rostrum is already highly elongated and ventrally curved compared to the smaller *Stenella* beak.

#### 3.2.3 Stage 18

CS 18 is defined in *Stenella* by digits II and III being equal in length. At this stage, the flipper takes on a hydrofoil shape and the digital rays are still cartilaginous with no evidence of joint formation. Our collection does not include *Delphinapterus* or *Balaena* specimens that display these combined features. This gap may suggest that variation in flipper development between *Stenella* and the other taxa predates this stage, given that both *Delphinapterus* and *Balaena* embryos already have asymmetry between digits II and III before this stage that is more prominent than the variation seen in *Stenella*.

#### 3.2.4 Stage 19

In *Stenella*, CS 19 is defined by a digit II that is longer than digit III and the emergence of a distinct beak (Thewissen & Heyning, 2007).

##### Delphinapterus

**Figure 4:**
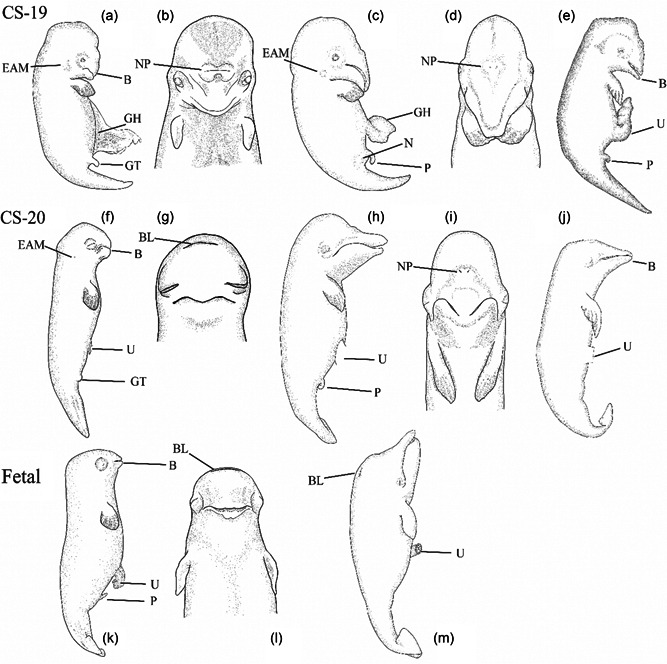
Embryos and fetuses of *Delphinapterus* (a, b, f, g, k, l), *Balaena* (c, d, h, i, m), and *Stenella* (e and j) for stages 19, 20, and fetal. All *Delphinapterus* and *Balaena* specimens shown in left lateral and frontal view. All *Stenella* specimens shown in left lateral view. (a and b) NSB‐DWM 2009LDL17F. (c and d) NSB‐DWM 2018G3F. (e) LACM 94743. (f and g) NSB‐DWM 2009LDL12F, (h and i) NSB‐DWM 2015B9F, (j) LACM 95014. (k and l) NSB‐DWM 2012LDL10F, (m) NSB‐DWM 2000B3F. B, beak; BL, blowhole; EAM, external acoustic meatus; GH, gut herniation, GT, genital tubercle; N, nipple; NP, nasal pits; P, penis; U, umbilicus. Not to scale.

In these embryos (NSB‐DWM 2009LDL17F, 2011LDL11F, 2009LDL9F), the forelimb is distinctly angular, transitioning from a rounded handplate to a flipper (Figure 4a). Digit II is both slightly longer and has more phalanges than digit III. There appear to be five phalanges in digit III. The umbilical hernia is still present, although reduced in comparison to younger specimens. The genital tubercle is present as a small protrusion. In all three specimens, the fluke shape is still lanceolate in appearance. The dorsal and ventral keels of the tail and flukes begin forming. One specimen (NSB‐DWM 2009LDL17F) has a prominent external acoustic meatus on the lateral aspect of the head (Figure 4b) while the other two specimens have small prominences posterior to the meatus. The eye is pigmented, and the eyelids are visible but not fused. A small beak is forming. The left and right nasal prominences are located near the midline and are separated by a thin strip of tissue.

##### Balaena

The *Balaena* forelimb is well‐developed, with obvious digit asymmetries and hyperphalangy (Figure 4c). Digit III is longer than digit II, with four phalanges in digit II and five phalanges in digit III. Specimen NSB‐DWM 2018G3F has visible paired protrusions next to the penis. This embryo also has moderate cervical flexion. The umbilical hernia of this specimen is almost entirely retracted into the body wall. The flukes are still diamond‐shaped in appearance, although they are markedly wider than in earlier CSs. There is a small process posterior to the external acoustic meatus (Figure 4d). The eyelids are visible and unfused. The two nasal pits are unfused and bilaterally symmetrical. The rostrum is elongated. There is no indication of either tooth or baleen formation.

##### Comparisons with *Stenella*

All features listed in *Delphinapterus* are also present in the *Stenella* embryos (Figure 4e) except for the phalangeal counts within the hyperphalangeous flippers. *Delphinapterus* phalangeal counts are 1/5/4/3/3. The *Stenella* embryos show a more extreme form of hyperphalangy in digit II, with phalangeal counts of 1/7/4/2/1. Additionally, though both odontocete taxa demonstrate emergence of the beak at this stage, the protrusion of the beak is less prominent in the *Delphinapterus* embryos than the *Stenella* specimens, consistent with postnatal morphology.

The *Balaena* specimen shows distinct heterochronic differences with the odontocetes. The eyelids and fluke morphology of NSB‐DWM 2018G3F suggest that this specimen is in stage 17 and the presence of hindlimbs in this specimen is comparable to a CS 16 *Stenella* or *Delphinapterus* embryo. However, digit III is longer than digit II in *Balaena*, in contrast to the defining characteristic of this stage for *Stenella*. NSB‐DWM 2018G3F shows a distinctly elongated rostrum, which would suggest that this embryo is in the CS 19 category. The ontogenetically younger *Balaena* embryo NSB‐DWM 1999B7F also has a long rostrum, indicating that this feature has appeared at an earlier stage. We place *Balaena* into this stage based on the exclusion of defining features for CSs 20, 21, 22, or 23, which are found in ontogenetically older *Balaena* embryos and fetuses.

#### 3.2.5 Stage 20

Stage 20 captures the transition from embryonic to fetal development in *Stenella*, as indicated by retraction of the umbilical hernia and fusion of the eyelids (Thewissen & Heyning, 2007).

##### Delphinapterus

In the *Delphinapterus* stage 20 specimens (Table 1), the forelimb has a clear flipper shape with prominent hyperphalangy (Figure 4f). Digit II and III are almost equal in length in some specimens, (NSB‐DWM 2017LDL3F, 2014LDL4F), although digit II is slightly longer than digit III for the other *Delphinapterus* specimens in this stage. Despite variation in digit length, digit II consistently has more phalanges than digit III. As one example, NSB‐DWM 2009LDL12F has eight phalanges in digit II and six phalanges in digit III. The embryos no longer exhibit significant cervical or lumbar flexion in the trunk and minimal ventral flexion at the tail base. Gut herniation is now fully retracted into the abdomen. The flukes change shape during this stage; smaller CS 20 specimens have a spade‐shaped fluke while the larger specimens have a heart‐shaped morphology. The fluke notch becomes apparent in larger embryos at this stage. Unlike previous stages, fluke width is greater than fluke length in the largest specimens (NSB‐DWM 2014LDL4F, 2014LDL5F) when measured from the fluke notch to the peduncle (the base of the fluke). Posterior to the external acoustic meatus is a small protrusion from the head (Figure 4g). Fusion of the two nasal prominences is apparent at this stage, with only one slit visible on the surface. The blowhole has migrated caudally in comparison to the earlier stages of *Delphinapterus* specimens and is located toward the crown of the head. The upper and lower eyelid are partially to fully fused.

##### Balaena

The forelimbs of both *Balaena* specimens (NSB‐DWM 1999B6F, 2016B9F) have an adult‐like flipper, with hyperphalangy and a hydrofoil shape (Figure 4h). Digit II has three phalanges, a reduction in phalangeal count by one compared to the CS 19 *Balaena* specimen (NSB‐DWM 2018G3F). Cervical flexure in these stage 20 specimens is greatly reduced, though the neck is still slightly bent. The umbilical hernia is fully retracted. These specimens have an elongated rostrum, eye pigmentation, and eyelids. The nasal pits are symmetrically paired at the midline (Figure 4i). The oral cavity and rostrum are longer than in stage 19. Baleen is not present in either specimen.

##### Comparisons with *Stenella*

The differences between *Stenella* (Figure 4j) and *Delphinapterus* are similar to the morphological disparities seen between postnatal specimens. The flippers of *Delphinapterus* are more rounded and mediolaterally wider than the pointed, narrow *Stenella* flippers. Additionally, the differences in beak shape observed in stage 19 are even more prevalent at stage 20. Both *Balaena* and *Stenella* specimens have elongated rostra. In contrast, the *Delphinapterus* specimens have a comparatively short beak. The flukes of *Stenella* at this stage are diamond‐shaped in smaller specimens while the largest CS 20 specimens have spade‐shaped flukes. In contrast, *Delphinapterus* have spade‐ and heart‐shaped flukes while the *Balaena* specimens have diamond‐ and club‐shaped flukes (Figure 8). At this stage, the flukes of *Stenella* are less developed than the flukes of the Arctic taxa.

*Balaena* at this stage have an elongated curved rostrum, which differs from the *Stenella* embryos. The nasal pits of both odontocete taxa are fused in the midline to form the blowhole, while the *Balaena* specimens maintain two distinct pits. This is a common feature of mysticetes and represents a divergence between the toothed and baleen whales (Klima, 1999).

#### 3.2.6 Fetal specimens

In the Carnegie system for *Stenella*, the differentiation between fetal stages 21, 22, and 23 are the emergence of tactile hairs, eyelid separation, and skin pigmentation, respectively (Thewissen & Heyning, 2007). It is important to note that the features defining fetal stages 21 and 22 emerge within a short developmental window. Thus, many *Stenella* are simply defined as CS 21/22 to accommodate the extensive overlap.

##### Delphinapterus

These *Delphinapterus* specimens (NSB‐DWM 2012LDL3F, 2014LDL7F, 2012LDL10F, 2019LDL11F, 2012LDL9F, 2016BDL3F, 2012BDL1F) cannot be assigned to stages 21–23 due to the lack of hairs, eyelid separation, or skin pigmentation (Figure 4k,l). The features that define these fetal stages in *Stenella* are not found in any of the largest *Delphinapterus* in our collection. These differences may be due to heterochronic shift of all three traits within these whales, or these features may occur in *Delphinapterus* fetuses that are developmentally older than the ones within our collection. All flipper and facial traits remain similar between stage 20 and these larger *Delphinapterus* specimens. Fetal specimen NSB‐DWM 2012LDL10F has seven phalanges within digit II and five phalanges for digit III, which is a reduction in one phalanx per digit compared to stage 20 *Delphinapterus* specimen NSB‐DWM 2009LDL12F. The other difference is a change in fluke shape (Figure 7). Although not described in Thewissen and Heyning (2007) as differential criteria, stages 20 and 21 *Stenella* embryos have differently shaped flukes. *Stenella* stage 20 specimens have heart‐shaped flukes while stage 21/22 specimens have triangular‐shaped flukes, suggesting that there is a transition in fluke shape through these fetal stages (Figure 7).

##### Balaena

*Balaena* fetuses are not significantly different in morphology between stages 20 and 21 (Figure 4m). One specimen in our collection represents stage 21, DWM‐NSB 2000B3F (Figure 4m). This specimen is nearly identical to the stage 20 *Balaena* specimens. The flipper is hyperphalangeous, with five phalanges on digit III. The flukes are more developed, achieving a triangular‐shaped morphology in stage 21, and the body has pigmentation. The major morphological transition left between this stage and birth is the development and emergence of baleen. Thewissen et al. (2017) classifies these fetal *Balaena* with baleen as stage 23 (NSB‐DWM 2007B16F, 2009KK1F, and 2015B9F). The animals listed in that study are approximately four times larger than this stage 21 *Balaena* fetus.

#### 3.2.7 Flipper size

**Figure 5:**
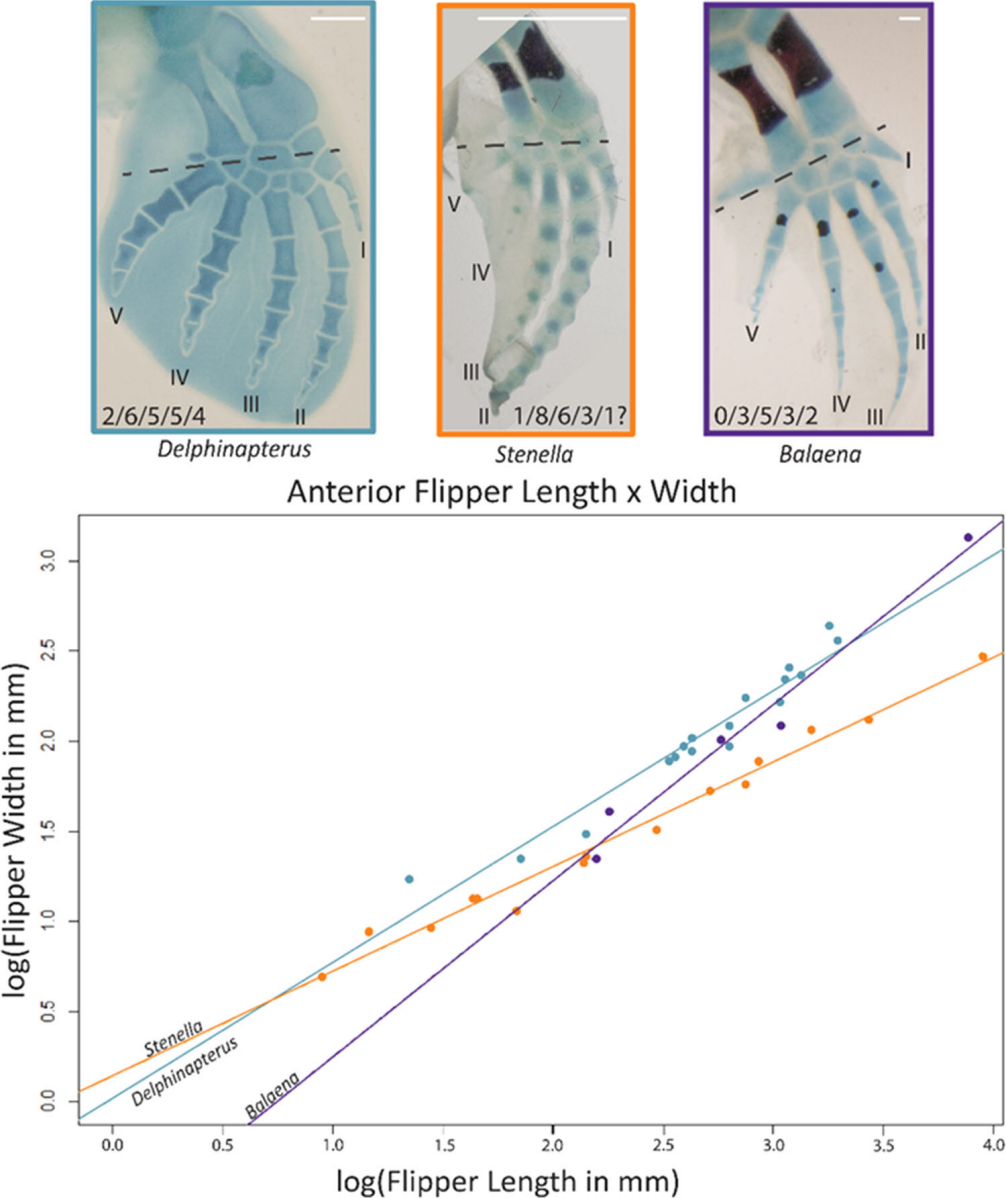
Flippers of CS 20 NSB‐DWM 2012LDL9F *Delphinapterus* (left, blue), CS 20 LACM 90310 *Stenella* (middle, orange), and CS 21 NSB‐DWM 2000B3F *Balaena* (right, purple). Cartilage is stained with alcian blue, and bone is stained with alizarin red. Graph shows relationship between anterior flipper length, from axilla to leading edge, and flipper width, taken at the midcarpal joint. Dashed lines on photo represent plane of measurement, digits indicated by roman numerals. Scale bar equals 0.5 cm

*Delphinapterus* and *Balaena* whale embryos display segmentation of their digital rays and hyperphalangy at an earlier CS than *Stenella*. At stage 17, both *Delphinapterus* and *Balaena* embryos have clearly defined digital rays and individual phalanges are readily identifiable (Figure 3d,f). Phalangeal segmentation is not observed in *Stenella* until CS 19 (Cooper et al., 2017), though stage 18 embryos show length asymmetries between digits II and III (Figures 3g and 4e). Despite the later onset of phalangeal segmentation in *Stenella*, this taxon has more phalangeal segments in the longest digit than either *Balaena* or *Delphinapterus* (Figure 5).

At CS 20, both Arctic taxa have 5‐6 phalanges in their longest digit ray, digit II in *Delphinapterus* and digit III in *Balaena* (Thewissen, Hillmann, George, Stimmelmayr, et al., 2021), while a similarly staged *Stenella* fetus has eight phalanges in digit II (Cooper et al., 2007, 2017) (Figure 5). As such, the *Stenella* flipper is proportionally longer proximodistally and anteroposteriorly thinner than the other fetal cetacean specimens at CS 20. *Delphinapterus* and *Balaena* have similar flipper growth trajectories despite differences between digit II and digit III as the longest digit. *Stenella*, having extreme hyperphalangy, has a growth trajectory that is is markedly different from the other cetaceans (Figure 5).

#### 3.2.8 Fluke size

**Figure 6:**
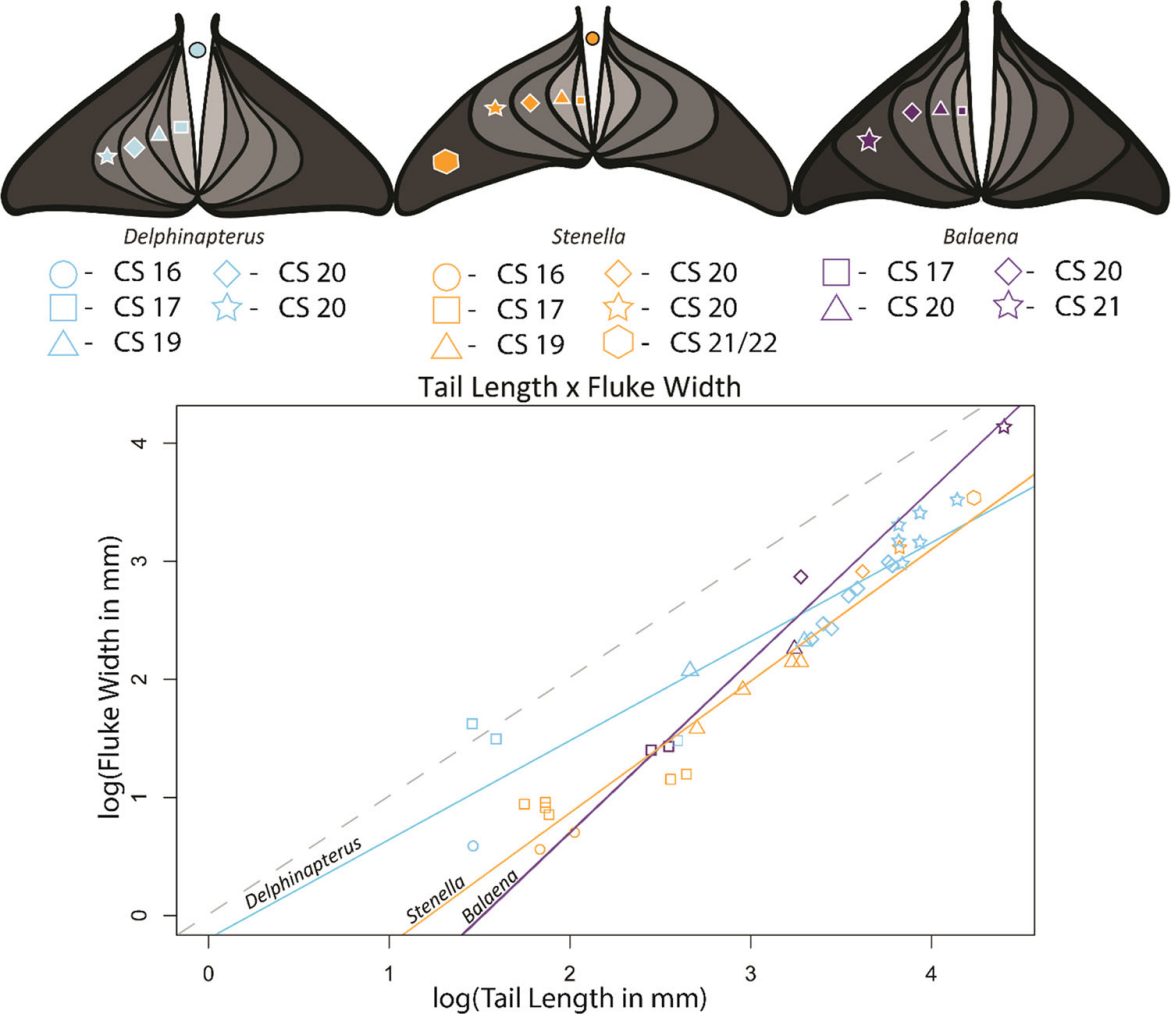
Diagrams of ontogenetic fluke growth morphologies for *Delphinapterus* (left), *Stenella* (middle), and *Balaena* (right). Shapes embedded within fluke illustrations correspond to intermediate growth morphology plotted on graph. Measurements, taken to the nearest millimeter, include tail length from genital tubercle to fluke tip, and maximum width of fluke. Dashed line shows isometry. Diagrams not to scale.

During embryonic development, all three taxa initiate fluke growth at approximately the same CS. Cetacean fluke growth proceeds from a small, diamond‐shaped outgrowth through a series of intermediate shapes before arriving at taxon‐specific morphologies. Our data suggest that *Delphinapterus* initiates fluke growth and then gradually increases fluke width relative to tail length, where tail length is defined by the distance from the genitals to the tip of the fluke notch (Figure 6). In contrast, *Stenella* has a more elongated tail relative to fluke width for the initial stages of fluke growth but attains rapid lateral fluke outgrowth in the early fetal period. *Stenella* is the only taxon to achieve an adult‐like fluke early in the fetal period. Both odontocete taxa initiate small lateral fluke outgrowths around CS 17 and then show a rapid transition from this diamond‐shaped morphology to a spade‐ or heart‐shaped intermediate morphology.

Fluke ontogeny for *Balaena* is not completely documented within specimens available to us, although several insights can be gleaned. At CS 20, the two specimens have nearly identical tail length but differently shaped flukes (Figure 6). DWM‐NSB 2016B9F has a total length of 122.6 mm, genital to fluke notch length of 24.8 mm, fluke width of 8.7 mm, and a spade‐shaped fluke. DWM‐NSB 1999B6F has a total length of 166 mm, genital to fluke length of 25.6 mm, a fluke width of 16.68 mm, and a heart‐shaped fluke. It appears that fluke growth increases with total body length, although the tail length remains relatively stable. While all three taxa initiate fluke outgrowth at CS 17, the taxon‐specific differences in lateral outgrowths become readily apparent (Figure 6).

## 4 DISCUSSION

### 4.1 Presomitic *delphinapterus* specimen (NSB‐DWM 2017LDL21F)

This specimen is ontogenetically younger than any staged *Delphinapterus* embryos. However, when compared to other cetacean embryos, NSB‐DWM 2017LDL21F is more developed than the *Balaenoptera acutorostrata* morula described in Asada et al. (2001), which may be one of the youngest cetacean embryos described.

The fetal membranes appear similar to the supporting membranes in humpback whale (*Megaptera novaeangliae*) embryos shown in Stump et al. (1960). Both our *Delphinapterus* specimen and the *Megaptera* supporting membranes referenced in Stump et al. (1960) are long and thin, with two projections from a centralized region where the embryo is contained. However, these specimens are remarkably different in structure despite similarities in fetal membrane morphology. The *Megaptera* specimen from Stump et al. (1960) already appears to be postsomitic whereas this *Delphinapterus* specimen shows no indications of somitogenesis. In a typical mammalian embryo at the gastrulation or neurulation stage, we would expect to find structures such as a primitive streak, notochord, or neural tube. However, none of these morphological characteristics are readily found in NSB‐DWM 2017LDL21F.

Given the unusual appearance of this specimen, we hypothesize that NSB‐DWM 2017LDL21F is an aberrant embryo that would have eventually been incompatible with life. There is considerable evidence that *Delphinapterus* have a synchronized mating schedule (Steinman et al., 2012). The narrow window of early development showcased by the embryos and fetuses collected from the harvests in Point Lay, Alaska, between CSs 16 and 21, are consistent with a constrained breeding season. Our presomitic specimen is clearly younger than other embryos collected from Point Lay, which further supports our conclusions that this embryo had likely ceased development prematurely.

Externally, the specimen showed clear bilateral symmetry with a number of structures resembling a potential stomodeum (Figure 1d) or notochord (Figure 1e). However, virtual segmentation and histological sectioning of the specimen do not reveal any cell types associated with these structures. Furthermore, there is a clear differentiation of some tissues within the histological sections, however, the relationships of these cell types do not indicate any organized organ primordia. For example, we see a potentially cardiogenic region based on the presence of red blood cells and mesenchymal tissue (Figure 2c). However, that morphology does not appear to match cardiac primordia from similarly staged pig embryos (Patten, 1931). Furthermore, at approximately this gestational age in humans, the embryo and fetal membranes are similar in size (O'Rahilly & Müller, 2001). In this specimen, the fetal membranes were several times larger than the embryo. This suggests that the development of this *Delphinapterus* embryo was arrested while the membranes continued to grow.

#### 4.1.1 Staging system

**Figure 7:**
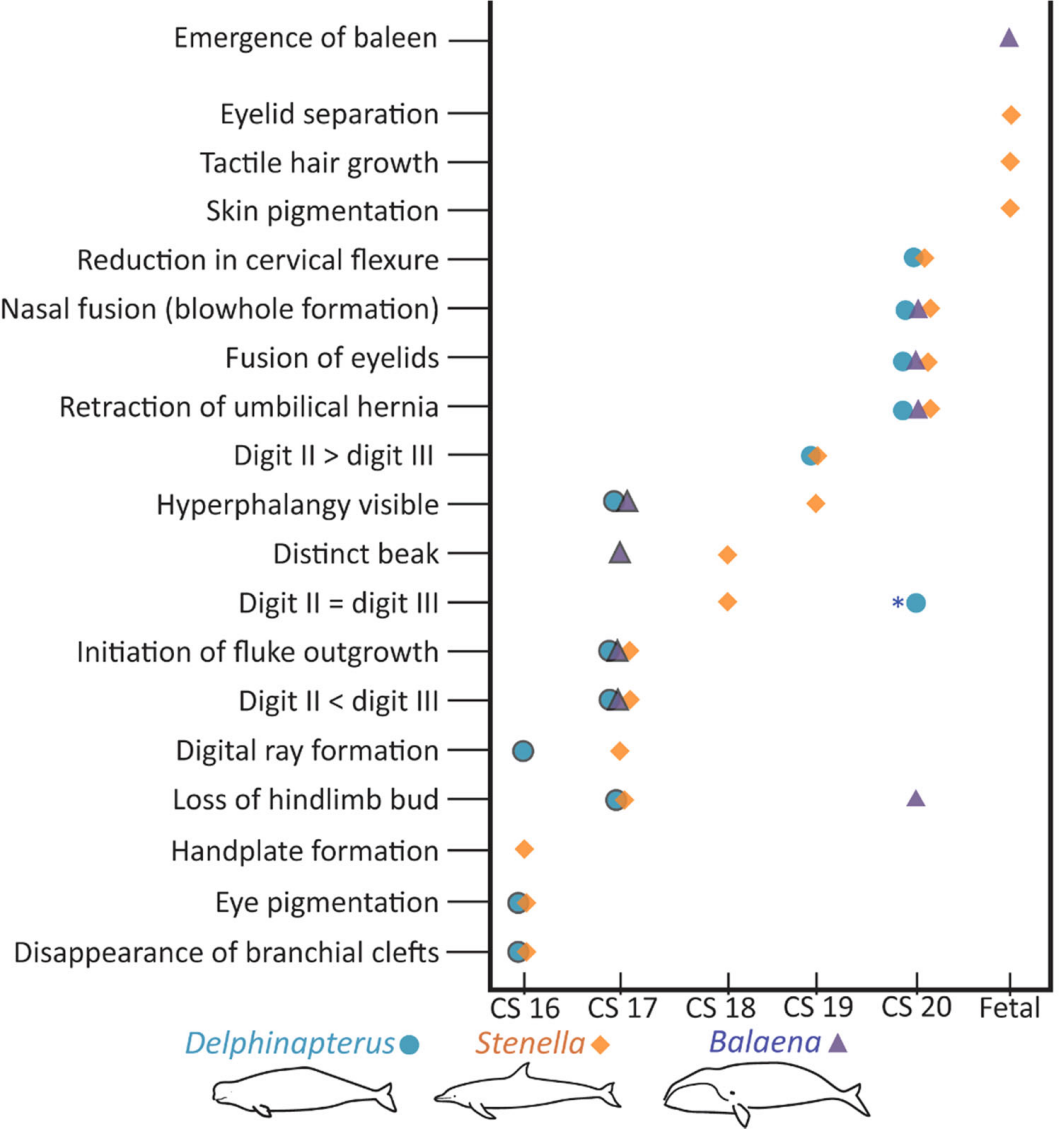
Heterochronic development between *Stenella*, *Delphinapterus*, and *Balaena*. Symbols indicate presence of specific characters within these taxa. Outlined points indicated traits identified in one specimen. If no point is shown that trait was not able to be identified in our collection either due to developmental variation or sampling bias. *Only occurs in some of the CS 20 *Delphinapterus* specimens, the flipper typically maintains digit II > digit II in both phalangeal count and absolute length.

Our analysis of cetacean embryos reveals developmental variation between *Delphinapterus*, *Balaena*, and *Stenella* (Figure 7). Some features, such as the blowhole, display embryonic timing differences between odontocetes and mysticetes. While the developing nasal cavities initiate as paired processes during development in all cetacean taxa, eventually the two nares will fuse in the midline in odontocetes, forming one blowhole. For *Balaena* and other mysticetes, the nares remain separated by a septum throughout life. There is a fair body of evidence suggesting that odontocetes and mysticetes have differential patterns of development that lead to two separate mechanisms of blowhole orientation within the skull (Armfield et al., 2011; Kellogg, 1928; Moran et al., 2011; H. A. Oelschläger, 1989; Roston & Roth, 2021).

**Figure 8:**
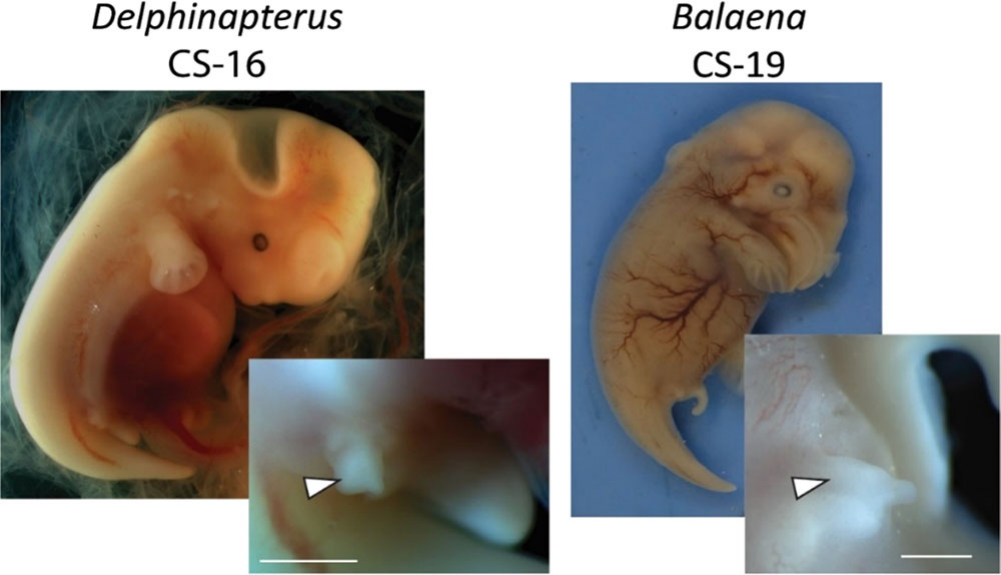
Photographs of CS 16 *Delphinapterus* (NSB‐DWM 2019LDL15F—left) and CS 19 *Balaena* NSB‐DWM 2018G3F—right) embryos. Hindlimbs (white arrows) are visible in both of these specimens. Scale bar equals 0.5 mm.

Other features, such as rostrum formation, do not follow systematic divisions and thus may represent areas of heterochronic development. Both *Balaena* and *Stenella* develop an elongated, prominent rostrum by CS 18, whereas in *Delphinapterus* the oral cavity remains comparatively short (Figure 7). The most prominentmorphological changes that contribute to the Carnegie staging system are within the flipper, hindlimb bud, and fluke.

#### 4.1.2 Flipper

The most prominent difference between these taxa is the variation in the longest digit of the flipper and the degree of hyperphalangy on each digit (Table 2).

**Table 2:** Summary of phalangeal counts for relevant Carnegie stages

	*Delphinapterus*	*Stenella*	*Balaena*
CS 16	Digital rays, no segmentation	Handplate	Unknown
CS 17	?/5/3/5/2	Digital rays, no segmentation	0/4/5/4/3
CS 18	N/A	Digital rays, no segmentation	N/A
CS 19	?/6/5/5/3?	1/7/4/2/1?	0/3/5/4/3
CS 20	2/6–8/5–6/5/4	1/8/6/3/1?	0/3/5/3/2
Adult	0–3/6–8/5–7/5–6/4–6	1/7–9/4–6/1–2/0–1	0–2/3–4/4–5/3–4/2–3

*Note*: Counts are read from anterior to posterior. Adult data for *Delphinapterus* (Kleinenberg, 1969; Kükenthal, 1889), *Stenella* (Cooper et al., 2017; Fedak & Hall, 2004; Sedmera et al., 1997b), and *Balaena* (Cooper et al., 2007; Fedak & Hall, 2004; Thewissen, Hillmann, George, Tarpley, et al., 2021) is published elsewhere. Question mark indicates uncertainty of phalangeal formula at that particular Carnegie stage.

The longest digit for *Delphinapterus* and *Stenella* is digit II; this digit also has the greatest number of phalanges in prenatal and postnatal individuals. In contrast, digit III is the longest digit in the mysticete *Balaena*. This variation in the longest digit length between odontocetes and mysticetes has been previously documented in postnatal individuals (Cooper et al., 2007). *Delphinapterus* has a hyperphalangeous phenotype on all digits except for the reduced digit I. In contrast, *Stenella* shows extreme hyperphalangy on digit II and hyperphalangy on digit III but reduced phalangeal numbers on digits IV and V compared to the mammalian standard of 2/3/3/3/3. Depending on the individual, a *Balaena* specimen will consistently have hyperphalangy of digit III, but may not have a hyperphalangeous phenotype on digits II or IV.

The adult *Delphinapterus* flipper phalangeal formula has a variable range of 0–3/6–8/5–7/5–6/4–6 (Kleinenberg, 1969; Kükenthal, 1889). The prenatal specimens mentioned in this study have phalangeal counts ranging 0–2/6–8/5–6/5/4. The adult *Balaena* phalangeal formula is 0–2/3–4/4–5/3–4/2–3 (Cooper et al., 2007; Fedak & Hall, 2004; Thewissen, Hillmann, George, Tarpley, et al., 2021). Prenatal *Balaena* from this study show a phalangeal count of 0/3–4/5/3–4/2, which matches the adult condition. In contrast to the Arctic whales, *Stenella* demonstrates a more hyperphalangeous phenotype. The adult phalangeal formula for *Stenella* is 1/7–9/4–6/1–2/0–1 (Cooper et al., 2017). This count is greater than the number of phalanges found in the prenatal specimens on digits II and IV, which have a formula of 1/7–8/4–6/2–3/1. In adult cetaceans, several interphalangeal joints can form, and phalanges can remain cartilaginous, which may contribute to the variation in counts between prenatal and postnatal specimens seen here. These variations in the number of phalanges and longest digit length are reflected in the growth trajectories of the flipper (Figure 5) and may be related to differences in gene signaling related to hyperphalangy.

Previous developmental work has experimentally shown that the generalized tetrapod hand is modified into a hyperphalangeous flipper via several critical modifications to the typical genetic cascade directing limb development. One such modification is the continued expression of the protein FGF8, which is critical for the maintenance of the apical ectodermal ridge (AER) that supports proximodistal growth of the embryonic limb. In comparison to a more typical mammalian limb found in mice, the *Stenella* forelimb shows prolonged FGF8 protein signaling, probably extending the length of the limb (Cooper et al., 2017). Additionally, WNT9A, an essential protein for phalangeal joint formation is expressed within the cetacean limb. Augmentation of this protein is implicated in the increased segmentation of digit primordia within *Stenella* (Cooper et al., 2017). While all extant cetacean taxa demonstrate hyperphalangy and probably have prolonged FGF8 and WNT9A signaling compared to terrestrial, pentadactyl mammals, it is possible that protein signaling for FGF8 and WNT9A is reduced in *Balaena* and *Delphinapterus* with respect to the extreme hyperphalangy found in *Stenella*, given that both arctic taxa have fewer phalanges than *Stenella*.

The cetacean flipper initially evolved from a pentadactyl, weight‐bearing limb in early, amphibious archaeocetes, and with a change in ecological niche came a drastic restructuring of the skeleton, including the manus and forelimb (Gavazzi et al., 2020). Early amphibious fossil whales are hypothesized to have partial interdigital webbing on the limbs (Gingerich et al., 2001, 2009, 2019; Lambert et al., 2019; Madar, 2007; Thewissen et al., 1996) By the emergence of *Dorudon*, the first pelagic archaeocete, the forelimb was completely encased in a soft‐tissue flipper (Uhen, 2004), though there is no evidence of hyperphalangy. Some of the earliest evidence for hyperphalangy is in the 7–8 MYA mysticete fossil *Balaenoptera siberi* (Pilleri, 1990), though this does not preclude the possibility of an earlier evolutionary timepoint. Thus, interdigital webbing found around the cetacean digits and the emergence of hyperphalangy are evolutionarily distinct events despite developmental integration.

#### 4.1.3 Hindlimbs

Extant cetaceans do not have externally visible hindlimbs; the structures formed by the temporary hindlimb buds are embedded within the body wall and the pelvis, which is not articulated to the vertebral column, supports the urogenital system. For the delphinoids, which include both *Delphinapterus* and *Stenella*, the hindlimb bud only forms a rudimentary pelvis and occasionally a cartilaginous femur (Amasaki et al., 1989; Sedmera et al., 1997a). In *Balaena* other elements of the hindlimb can also form, with both femora and tibiae present in the body wall (Struthers, 1881; Thewissen, Hillmann, George, Tarpley, et al., 2021).

Both *Delphinapterus* and *Stenella* maintain external hindlimb buds through CS 16 (Figure 8). The *Stenella* hindlimb buds regress fully by stage 17 and are no longer visible externally. It is unclear if *Delphinapterus* retains hindlimb buds into stage 17; there is no evidence of hindlimbs in NSB‐DWM 2013LDL6F, though the abdominal wall is damaged. In contrast, *Balaena* embryos retain hindlimb buds for a prolonged period and the hindlimb buds clearly persist through stage 19 (Figure 8). The hindlimb bud of NSB‐DWM 2018G3F (CS 19) is elongated with a small epithelial protrusion on the end which may be an indication of an AER, the genetical signaling center for limb outgrowth.

Developmentally, the persistent presence of an AER, the region which drives proximodistal limb growth, and the associated signaling factors, would promote continuous cellular differentiation that led to the development of more elaborate hindlimb structures (Richardson et al., 2004, 2009; Tabin & Wolpert, 2007). In *Stenella*, cessation of SHH signaling from the zone of polarizing activity, a region that determines anteroposterior limb asymmetries, is implicated in the truncation and termination of hindlimb development (Thewissen et al., 2006). Because *Balaena* embryos clearly demonstrate the prolonged presence of hindlimb buds in comparison to other cetacean taxa, it is likely that this taxon extends the timeframe of AER expression, allowing for the formation of more hindlimb structures compared to other cetaceans. Furthermore, given that hindlimb loss has likely only evolved once in the cetacean lineage (Sedmera et al., 1997a; Thewissen et al., 2006; Uhen, 2004), this aberrant SHH signaling found in *Stenella* is possibly involved in hindlimb truncation in all of Cetacea, though this hypothesis has yet to be tested empirically in other taxa.

Early amphibious archaeocetes primarily relied on hindlimb‐dominated swimming via pelvic paddling or pelvic oscillation (Bebej & Smith, 2018; Thewissen & Fish, 1997). The pelvis was fully articulated to the vertebral column in early fossil cetaceans like *Ambulocetus*, *Remingtonocetus*, and many protocetids (Bebej et al., 2012; Gingerich et al., 2001, 2009; Thewissen et al., 1994; Uhen, 2014). There is evidence that some protocetids, such as *Aegicetus* and *Georgiacetus*, lack sacroiliac articulation (Gingerich et al., 2019; Hulbert, 1998; Uhen, 2008), however, both of these animals still retained elaborate, functional hindlimbs. *Dorudon* and *Basilosaurus*, in contrast, had greatly reduced hindlimbs compared to their overall body size. Their hindlimbs did not articulate to the vertebral column and probably did not serve a functional role during locomotion (Gingerich et al., 1990; Uhen, 2004).

#### 4.1.4 Fluke

All modern cetaceans have lunate‐shaped flukes, the main organ necessary for propulsion during locomotion. All three taxa analyzed here initiate fluke development at approximately the same CS. However, the growth rates of the flukes vary between all three species, and taxon‐specific variation quickly becomes apparent. The postnatal shape of the flukes is tailored for efficiency during swimming behaviors related to locomotion and feeding (Ayancik et al., 2020).

The genetics of cetacean fluke development are currently not understood. It has been hypothesized that flukes share common protein signaling with limb buds (Infante et al., 2018; Thewissen, 2018). However, no data supporting or refuting this hypothesis have been published. Elucidating the mechanisms of fluke outgrowth in one taxon would likely be applicable to both extant and fully aquatic fossil cetaceans given that flukes likely developed once within the evolution of Cetacea, concurrent with the reduction and internalization of the hindlimbs.

The evolution of the flukes cannot be tracked directly, as soft tissue is not preserved within the fossil record. Instead, the development of the flukes within the archaeocetes is investigated via skeletal traits. Uhen (2004) proposed that fluke presence is correlated with the presence of a ball vertebra, which is convex on both the proximal and distal vertebral bodies. It is found in the first fully aquatic basilosaurid, *Dorudon atrox* (Uhen, 2004) but tails for many earlier cetaceans are not well preserved.

## 5 CONCLUSION

Our objective was to compare the early embryology of *Delphinapterus* and *Balaena* to the previously described *Stenella*. While *Delphinapterus* generally follows the *Stenella* based staging system with a few notable variations, identification of early *Balaena* exposes heterochrony between odontocetes and mysticetes. The two smallest *Balaena* specimens in our collection, NSB‐DWM 1999B7F and NSB‐DWM 2018G3F display hyperphalangy (a CS 19 trait for *Stenella*), hindlimb buds (CS 16), elongate rostra (CS 19), and small fluke outgrowths (CS 17). Thus, trying to bin these mid‐to‐late embryos into the existing Carnegie staging system proves challenging. Some CSs, such as CS 18, which is defined by a digit II = digit III, are not applicable for any of the *Balaena* specimens that we examined and only further highlight the differences in morphology observed between the odontocetes and mysticetes. Lastly, the heterochrony among bowhead whale embryos compared to the odontocetes also has implications for fetal growth, which is used for inferences about gestation length and mating period in *Balaena* (e.g., Christiansen et al., 2022; Reese et al., 2001). Though embryonic length measurements are not an integral component of the Carnegie staging system, our limited bowhead data set illustrates overlap in morphological features at variable embryo length (see also Thewissen, Hillmann, George, Tarpley, et al., 2021). Further research is needed to resolve developmental chronology and associated length variation for mid to late bowhead whale fetuses.

Some of the most striking morphological changes during cetacean development center around the flippers, hindlimbs, and flukes. *Delphinapterus* and *Balaena* flippers are similar to each other morphologically while *Stenella* demonstrate a more extreme form of hyperphalangy. Hindlimb bud development is exceptionally prolonged in *Balaena* compared to the odontocete taxa. The hypermorphosis of the hindlimb bud in *Balaena* compared to the odontocetes is directly reflected in the extensive postnatal skeletal morphology. The flukes of all three taxa initiate around the same CS. However, the growth patterns vary. *Delphinapterus* flukes grow at a consistent pace, whereas *Stenella* flukes appear to transition between intermediate fluke shapes quickly, especially between a spade‐shaped morphology and the adult‐like fluke. The *Balaena* fluke growth trajectory has the steepest growth rate of any taxon based on our current, albeit incomplete, data set.

Our paper is an attempt to commence a developmental staging system for all of Cetacea. This work forms the basis for understanding heterochrony in these highly derived mammals. The integration of cellular and molecular data will further enrich our understanding of cetacean ontogeny and evolution.

## Data Availability

The data that support the findings of this study are available from the corresponding author upon reasonable request.
